# Successful transcatheter arterial embolization for pseudoaneurysm of the deep femoral artery in a patient with presumptive ACTA2-related vasculopathy

**DOI:** 10.1016/j.radcr.2021.09.007

**Published:** 2021-10-01

**Authors:** Satoru Kamio, Takatoshi Kubo, Saori Koshino, Osamu Abe

**Affiliations:** Department of Radiology, The University of Tokyo Hospital, 7-3-1 Hongo, Bunkyo-ku, Tokyo 113-8655, Japan

**Keywords:** Acta2, Acta2-Related Vasculopathy, Pseudoaneurysm, Transcatheter Arterial Embolization

## Abstract

ACTA2-related vasculopathy is an autosomal dominant genetic disorder characterized by aortic aneurysms and dissection, and limb artery lesions are rare. We report a case of transcatheter arterial embolization for a pseudoaneurysm of a deep femoral artery in a patient with presumptive ACTA2-related vasculopathy. A 58-year-old woman was presumed to have an *ACTA2* mutation based on her history of aortic diseases and family history of *ACTA2* mutations. During follow-up, contrast-enhanced computed tomography for aortic diseases revealed occlusion and vessel wall abnormalities of the bilateral deep femoral arteries. Two weeks later, she complained of acute right inguinal pain without any triggering factors, and contrast-enhanced computed tomography revealed a pseudoaneurysm of the right deep femoral artery. Vascular fragility due to *ACTA2* mutation was believed to be the cause of the pseudoaneurysm. Transcatheter arterial embolization was successfully performed and no rebleeding occurred during 1.5 years after the transcatheter arterial embolization.

## Introduction

ACTA2-related vasculopathy is an autosomal dominant genetic disorder characterized by aortic aneurysms and dissection [1]. ACTA2-related vasculopathy is mainly associated with thoracic aortic lesions, and limb artery lesions are rare [2]. There has been no report of transcatheter arterial embolization (TAE) for ruptured limb arteries due to ACTA2-related vasculopathy. Here, we report a case of TAE for a pseudoaneurysm of a deep femoral artery (DFA) in a patient with presumptive ACTA2-related vasculopathy.

## Case Report

A 58-year-old woman had a history of aortic root dilatation, ascending aortic aneurysm, and patent ductus arteriosus, and had undergone aortic root and ascending aortic replacement and closure of the ductus arteriosus. Her mother, brother, and sisters had a history of aortic dissection, and her brother was identified as having an *ACTA2* mutation. *ACTA2* mutation testing was not performed on the patient's request, but based on her history and family history, the patient was presumed to have ACTA2-related vasculopathy.

**Fig. 1:**
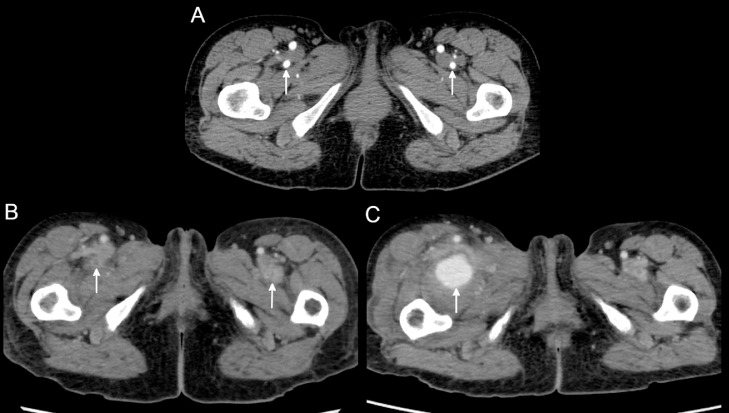
Contrast-enhanced computed tomography (CT) at the level of the deep femoral artery (DFA). (A) Contrast-enhanced CT showing normal bilateral DFAs (arrows). (B) Contrast-enhanced CT performed 1 month after (A) showed occlusion and abnormal vessel wall thickening of bilateral DFAs (arrows). (C) Contrast-enhanced CT performed 2 weeks after (B) showed a pseudoaneurysm of the right DFA (arrow).

A follow-up contrast-enhanced computed tomography (CT) for aortic disease showed abnormal wall thickening, dilatation, and occlusion of bilateral deep femoral arteries during the course of one month (Figs. 1A and B). Two weeks later, she complained of sudden right inguinal pain and swelling without any triggers, and contrast-enhanced CT showed a 35 mm pseudoaneurysm of the right DFA (Fig. 1C).

**Fig. 2:**
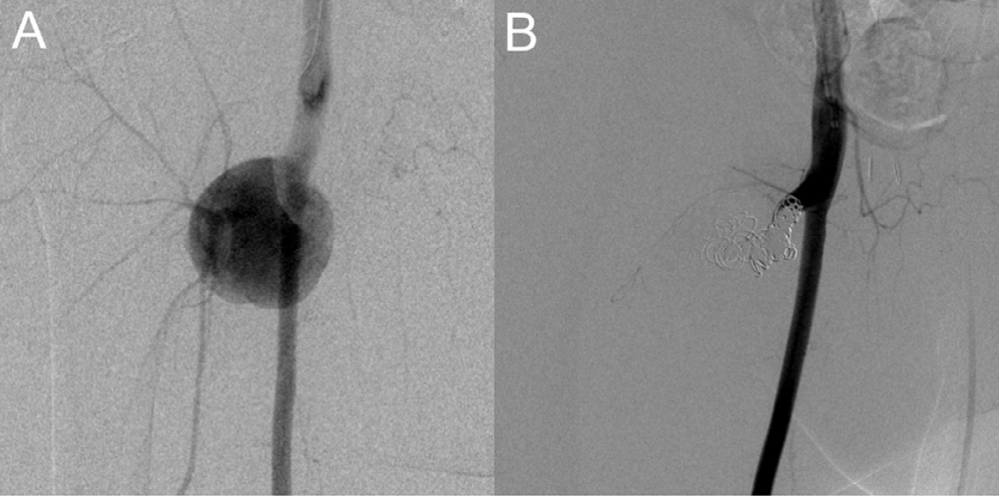
Digital subtraction angiography (DSA) of transcatheter arterial embolization (TAE). (A) DSA showed a pseudoaneurysm at the root of the right deep femoral artery (DFA) with distal occlusion of the DFA. (B) There was no residual pseudoaneurysm after coil embolization.

The pseudoaneurysm was treated with TAE. A sheath was inserted through the left common femoral artery, and a 4Fr shepherd's hook catheter (Medikit, Tokyo, Japan) was advanced into the right DFA. Digital subtraction angiography (DSA) showed a pseudoaneurysm at the bifurcation of the right lateral femoral circumflex artery in the right DFA, and the distal right DFA was occluded (Fig. 2A). A microcatheter (Renegade, Stryker, Michigan, USA) was inserted into the right lateral femoral circumflex artery and the back door of the pseudoaneurysm was embolized with pushable coils (Tornade, COOK medical Japan, Tokyo, Japan) and detachable coils (Interlock, Boston Scientific Corporation, Marlborough, Massachusetts, USA). Because of the rapid blood flow and wide neck of the pseudoaneurysm, a 5.2Fr balloon catheter (Cerecon MP, Terumo, Tokyo, Japan) was inserted into the root of the right DFA for flow control. Under dilatation of the balloon catheter, the front door of the pseudoaneurysm was embolized with pushable and detachable coils. After embolization, DSA confirmed the disappearance of the pseudoaneurysm and the patency of the right superficial femoral artery (Fig. 2B). No rebleeding occurred in the 1.5 years after the TAE.

## Discussion

The *ACTA2* gene (OMIM #102620, ORPHA 91387, 2573, 404,463) encodes the smooth muscle cell isoform of alpha-actin, an important component of the vascular smooth muscle contractile mechanism [1]. Mutations in *ACTA2* are the most frequently encountered cause of non-symptomatic heritable thoracic aortic disease, with a reported detection rate of 1.5%–21% [3].

ACTA2-related vasculopathy is an autosomal dominant inherited disease with the main phenotype of aortic aneurysm and aortic dissection caused by the *ACTA2* gene mutation. Previous reports have shown that the penetrance is incomplete and age-related, and the occurrence of aortic disease in children is rare [3]. In addition to aortic disease, a variety of other vasculopathy occur, including coronary artery disease, patent ductus arteriosus, pulmonary hypertension, moyamoya disease-like cerebral vascular stenosis, occlusion, and dilatation [1]. Vascular smooth muscle dysfunction and smooth muscle cell proliferation in the arterial intima and tunica media have been suggested to be involved in this vasculopathy [1].

In this case, ACTA2-related vasculopathy was presumed based on the patient's history and family history. Abnormal wall thickening, dilatation, and occlusion of bilateral deep femoral arteries, and rupture of the right DFA appeared within a short time without any triggers, which was thought to be caused by presumptive ACTA2-related vasculopathy. There are few reports of limb arterial lesions caused by ACTA2-related vasculopathy, and only axillary aneurysms have been reported in multisystem smooth muscle dysfunction syndrome, a severe phenotype of ACTA2-related vasculopathy caused by Arg179 mutation in the *ACTA2* gene [4]. To the best of our knowledge, there have been no reports of DFA lesions.

This is the first case of a ruptured limb artery caused by presumptive ACTA2-related vasculopathy treated with TAE. Although there is no consensus on the treatment of limb artery lesions caused by ACTA2-related vasculopathy, this case was successfully treated by TAE without recurrence for 1.5 years. TAE may be a treatment option for ACTA2-related vasculopathy, but further accumulation of cases is required to confirm the usefulness of TAE.

## Patient consent

Written informed consent for the treatment was obtained from the patient.
